# Posttraumatic Stress Disorder Content on TikTok: Cross-Sectional Analysis of Popular #PTSD Posts

**DOI:** 10.2196/71209

**Published:** 2025-09-10

**Authors:** Brittany Rohl, Laura Carolyn Jones, Rachel Nattis, Robert Dale Claar, Xavier Velez, Joy Gabrielli, John Williamson, Eric Porges

**Affiliations:** 1 Clinical and Health Psychology, College of Public Health and Health Professions, University of Florida, 1225 Center Drive, Gainesville, FL, 32610, United States, 1 (352) 273-6617; 2 Department of Psychiatry, College of Medicine, University of Florida, Gainesville, FL, United States

**Keywords:** posttraumatic stress disorder, TikTok, social media, health communication, PTSD

## Abstract

**Background:**

TikTok became an increasingly popular platform for mental health discussions during a major global stressor (COVID-19 pandemic). On TikTok, content assumed to promote user engagement is delivered in a hyperindividually curated manner through a proprietary algorithm. Mental health providers have raised concerns about TikTok’s potential role in promoting inaccurate self-diagnoses, pathologizing normal behaviors, and fostering new-onset symptoms after exposure to illness-related content, such as tic-like movements linked to conversion or factitious disorders. The accuracy of posttraumatic stress disorder (PTSD)–related content with respect to conveying symptoms, diagnosis, and treatment deserves further investigation.

**Objective:**

This study aimed to characterize the accuracy of PTSD-related TikTok content.

**Methods:**

In February 2022, a search was conducted on TikTok using the hashtag #PTSD, and the 100 most-liked videos were retrieved. Videos were excluded if they were in a non-English language, duplicated, unrelated to PTSD, lacked audio, or contained nonfunctioning links. A publicly available Python package (TikTokPy) was used to scrape available metadata (views, shares, etc). Using the Patient Education Materials Assessment Tool for Audiovisual Materials (PEMAT-AV), videos were independently coded by 2 reviewers for the overall accuracy of the video (useful, personal experience, or misleading), whether the creator self-identified as a health care professional, symptoms mentioned, and overall video understandability and actionability. A third reviewer was consulted in the rare instances of coding disagreements.

**Results:**

Of the 100 included videos, 29 were classified as useful, 59 were classified as personal experience (subjective experience without outright inaccuracies), and 12 were classified as misleading. The degree to which PTSD-related information was accurate was not associated with its understandability, actionability, or user engagement. Besides useful videos being longer (mean 88.7, SD 63.1 seconds) than personal experience videos (mean 42.7, SD 44.5 seconds), no group differences in video metadata were observed across the number of views, likes, shares, or comments (*P*>.05). While self-identified HCPs were more likely to post useful content, they also contributed to 33% (4/12) of misleading videos. Changes in cognition and mood were the most frequently reported symptoms of PTSD (38/100, 38% of videos).

**Conclusions:**

Our findings were roughly consistent with previous studies of mental health–related TikTok content accuracy, although this is variable by diagnosis. TikTok’s continuously adaptive algorithmic content delivery may expose users to nonspecific and potentially misleading “click-bait” mental health information, which could influence symptom interpretation and clinical presentation. Clinicians should be aware of the digital landscape shaping patients’ perceptions of PTSD.

## Introduction

TikTok (ByteDance) is a short-form, video-based social media platform that surged in popularity during the COVID-19 pandemic and currently has more than 1 billion active users worldwide. TikTok joins a long history of internet-based platforms that serve as media for sharing health-related information. Preliminary work describing content related to posttraumatic stress disorder (PTSD) on TikTok suggests that there are 2 primary themes: instructional videos (21.6%) and the sharing of personal stories (67.3%) [1]. The authors cautioned that in the context of minimal trigger warnings on potentially upsetting and harmful first-person content, PTSD-related videos on TikTok may pose a risk for health misinformation and possibly vicarious traumatization. The aim of this study was to report the first systematic assessment of information quality within PTSD-related TikTok content.

The format of TikTok’s platform may make it particularly vulnerable to the spread of misinformation. Content is delivered to each user’s homepage (“For You Page” or FYP) in an algorithmically determined, hyperindividually curated manner, with nearly all delivered content having been produced by relative strangers. This contrasts with other social media platforms in which users tend to play a more active role in curating their content feeds (ie, by choosing profiles to “friend” or “follow”). Although the precise algorithms used to drive content delivery on TikTok are proprietary and not publicly known, they are assumed to maximize the likelihood of user engagement (time spent viewing each video, “liking” and sharing content, and conversion of exposure to a “following”). These metrics are assumed to shape subsequent content delivery with each swipe from video to video, resulting in the experience of being funneled into easily permeable communities that appear to cluster around shared ideas, experiences, traits, and observations, among other characteristics [2]. As the application and its algorithms “learn” about the user’s preferences, it can come to represent an “algorithmized self,” in which individual characteristics are identified without the person having ever searched for related terms [3,4].

This landscape poses two major challenges to accurate dissemination of mental health–related content specifically: (1) the sensitivity and specificity with which niche content is delivered to appropriate users, and (2) the degree to which delivered content reflects scientific consensus about particular disorders and their treatment. For example, it is plausible that the algorithm could identify a user’s generalized distress, offer content related to a handful of psychological phenomena, and derive future content delivery via algorithmic personalization based on the characteristics of their engagement. Assuming some variability in the reasons why someone might engage with a piece of content, engagement and subsequent narrowing of algorithmically derived content feeds may not necessarily correspond to the accuracy, applicability, or appropriateness of the content for the particular user. Importantly, this raises questions about whether vulnerable individuals are at particular risk of misinformation.

Three authors of this paper (BR, LCJ, and RN) observed that symptom misattribution is particularly fraught with respect to posttraumatic stress, often termed “trauma responses” in online discourse. According to publicly available Google Trends data [5], searches for the phrase “trauma response” nearly doubled in late 2019, more than tripled by August 2020, and increased 900% by Fall 2022 compared to pre-2019 averages. Similar but relatively smaller increases were observed in searches for the term “trauma,” while longtime upward trends in searches for “PTSD” maintained their relative trajectory since such data began collection in 2004. The proliferation of media to meet the demand for trauma-related information reflected both factual information as well as misinformation, including the misclassification of vague and banal phenomena as related to trauma. A 2021 article in the popular internet-based magazine *Slate* reports, “The trend of trauma-ifying common behaviors is so pervasive that there are now viral jokes about it… …[the trend] is relaying a vast assortment of relatable annoyances and pitfalls of being human while simultaneously upping the stakes by saying, ‘We’re all this way because we are traumatized’” [6]. An epitomical TikTok by user @thebrrashmoron describes it best: “Hitting snooze more than once is a sign of four mental illnesses! I heard that from a TikToker with no degree or qualifications” [7].

Clinical mental health providers have raised concerns that TikTok use may be associated with inaccurate self-diagnoses, pathologization of normal experiences, and the propagation of new-onset symptoms seemingly acquired after viewing illness-related content (particularly, tic-like movements), which may represent sociogenic expressions of conversion or factitious disorders [8,9]. A study of TikTok content related to attention-deficit hyperactivity disorder (ADHD) reported that, of the most-popular videos using the hashtag #ADHD, 52% were classified as misleading with respect to symptomatology, diagnosis, or treatment, defined as being beyond typical variability in subjective patient experience, and involving factually incorrect information [10]. Examples of misleading content included assertions that “anxiety shivers,” “random noise-making,” and a “lack of object permanence” are “little known” symptoms of ADHD.

This study aims to assess the accuracy of TikTok content classified under the hashtag #PTSD. We chose #PTSD for several reasons. The first was due to the relative ease with which we could verify claims (ie, comparison to diagnostic criteria and studies using diagnostically confirmed patient groups). Second, the use of #PTSD rather than #traumaresponse allowed for clear expectations with respect to the timeframe of symptom onset and what constitutes a traumatic event. This may reflect a more clinically useful framework if attempting to generalize results to patient populations. Finally, the classification of symptoms across a threshold of “legitimacy” as a means of defining group membership may be an important component of online behavior in patients with posttraumatic stress [11].

We hypothesized that user engagement would favor unique or highly salient information, such that videos classified as misleading or as reflecting subjective experiences—not incorrect but based on individual perspectives—would receive higher engagement across all measures (likes, views, shares, and comments) compared to videos classified as wholly accurate. Due to perceptions of credibility, we hypothesized that videos posted by self-identified health care professionals (HCP) would receive higher engagement across all metrics as compared to creators who did not self-identify as HCPs (non-HCP). Finally, we hypothesized that information usefulness and HCP status would predict the degree to which information was effectively communicated.

## Methods

### Overview

This study was preregistered as an exploratory observational investigation with Open Science Framework.

### Sample Size

The sample size of 100 TikTok videos was chosen as a feasible and representative sample size to capture the most viewed videos on the platform. Previous studies have used similar sample sizes to analyze health-related videos on social media platforms such as TikTok and YouTube [10,12-15].

#### Search and Selection Procedures

On February 20, 2023, a new TikTok account was created for the purpose of this study using the desktop version of TikTok. The use of this study-specific account (occurring off-campus and using a nonuniversity-owned computer) was intended to avoid the influence of individual user data on the video retrieval algorithm. New TikTok accounts are prompted to choose a number of general interest categories to determine initial homepage offerings; however, we circumvented these influences by opting to skip this step. On the same day the account was created, the search function was queried with the hashtag “#PTSD.” On TikTok, as on many internet platforms, hashtags serve to both categorize videos and to assist algorithms in determining content that might be of interest to users [16].

To provide a buffer in anticipation of excluding some of the retrieved videos based on eligibility criteria, the first 200 results under the “Top” tab (suggesting that results are most-suggested by TikTok’s proprietary algorithm) were captured as hyperlinks, and 2 authors with expertise in Python (RDC and XV) applied an open-source data scraper called TikTokPy [17] to collect video metadata (ie, number views, likes, and shares). A similar approach was used in a previous study of the accuracy of health-related information on TikTok [10]. The Python package used in this previous study (TikTok Scraper [18]) was attempted but did not work, likely owing to coding changes in TikTok itself. The resulting dataset was sorted in descending order by view count, and inclusion and exclusion criteria were applied until 100 videos were determined to be eligible for further analysis.

Videos were included if they mentioned or implied content related to PTSD, including but not limited to etiology, symptomatology, diagnosis, lived experience with PTSD, witnessing PTSD symptoms in others, or PTSD management strategies. Exclusion criteria included non-English videos (n=0), videos completely unrelated to PTSD (n=10), duplicate videos (n=1), videos without audio (n=3), or those with nonfunctioning links suggesting that the video had been removed from the platform (n=5). A total of 19 of the initial 200 videos were excluded before the target sample of 100 was reached.

### Rater-Coded Variables

Two raters with clinical research experience related to PTSD (LCJ and RN) independently assessed all 100 videos using the Patient Education Materials Assessment Tool for Audio Visual Materials (PEMAT-AV) [19]. The PEMAT-AV is a reliable tool designed to evaluate the clarity and effectiveness of audio-visual patient education videos and is designed to assess their comprehensibility and usefulness. Of note, the PEMAT-AV has traditionally been applied to patient education materials developed by health professionals. Therefore, to help contextualize ratings, support internal consistency by assessors, and in line with other studies applying this measure to social media videos, raters took notes about each video’s content and style [10,20,21]. These notes were not systematically analyzed for this study but select descriptions are provided for contextual purposes. The PEMAT-AV instrument generates an overall total score as well as subscales pertinent to understandability and actionability, all expressed as percentages of total possible points. Per PEMAT-AV rating protocol, understandability was scored based on the clarity of the content’s purpose, word choice and style, organization, design, and use of visual aids. Actionability was scored based on whether the content identifies actions the user can take, breaks down any actions into manageable and explicit steps, and addresses the user directly when describing actions.

Consistent with previous studies of health information on TikTok, additional analysis included classification of video content into one of the following categories: (1) useful, (2) personal experience, or (3) misleading. Useful videos presented entirely accurate information about PTSD prevention, diagnosis, prevention, prognosis, symptoms, and treatments. It is important to note that descriptions of nonspecific symptoms, as long as they are not inconsistent with PTSD, were not necessarily grounds for removal from this category. Personal experience videos presented anecdotal information related to PTSD diagnosis, etiology, symptoms, or treatment that was not inconsistent with diagnostic criteria, research, or generally accepted clinical practice. Misleading videos contained speculative information that was uncorroborated by diagnostic criteria, research, or generally accepted clinical practice. Importantly, videos that were mostly factual or focused on personal experiences could be classified as misleading if they contained any factually incorrect information. Where appropriate, the *Diagnostic and Statistical Manual of Mental Disorders, Fifth Edition* (*DSM-5*) criteria for PTSD were applied to determine the consistency of presented symptoms with diagnostic criteria [22]. In the rare event of a disagreement, a third rater (BR) resolved disagreements between the other two raters, which occurred rarely. Representation of trauma types, *DSM-5* diagnostic criteria, and the video’s tone (meme or joke, educational, personal, or myth-debunking) were also coded.

### Statistical Analysis

Extreme values in metadata were expected given that the phenomenon of “virality” among highly engaged social media content is defined as substantially higher engagement than “nonviral” content. While extreme values in metadata may not represent “average” PTSD-related TikTok content, exceptionally high engagement offers important clues about its relevance to users, their reactions, and the degree of dissemination. Therefore, videos determined to be exceptional on any user engagement metric (3 or more SDs above or below the overall mean were removed for separate qualitative analysis; n=3). Resulting distributions were converted to *z* scores for ease of comparison across metrics but remained highly nonnormal (Table 1). Therefore, incremental power-based transformations were applied until normality was achieved, with all requiring Blom transformation [23]. Previous studies assessing highly skewed distributions of social media metadata have also used power-based transformations that preserve rank order [24]. As a result of these transformations, group analyses are interpreted with respect to the significance and magnitude of difference, but not absolute difference between the original metrics.

**Table 1.** Top 100 #PTSD TikTok video characteristics by usefulness group (*z* scores; N=100).

Characteristics	Misleading (n=12), mean (SD)	Personal experience (n=59), mean (SD)	Useful (n=29), mean (SD)	*P* value
Video length	0.13 (0.83)	−0.21 (1.0)	0.71 (0.87)	.01^a^
Views	−0.04 (0.82)	0.10 (1.10)	−0.38 (0.70)	.17
Likes	−0.07 (0.84)	0.14 (1.07)	−0.48 (0.79)	.13
Shares	−0.09 (0.80)	0.06 (1.04)	−0.06 (1.21)	.80
Comments	−0.09 (0.91)	0.08 (1.0)	−0.16 (1.14)	.63

a *P*<.05

To compare groups defined by usefulness ratings (useful, personal experience, and misleading) across variables measuring user engagement (likes, shares, and comments), one-way ANOVAs were conducted, with Welch F correction applied when assumptions of heterogeneity were not met. In the cases where a significant group effect was observed, Student *t* post hoc tests were used, unless nonhomogeneity of variance required the use of nonparametric Games-Howell post hoc test. Nonparametric Kruskal-Wallis tests were used to assess PEMAT-AV understandability and total scores due to nonnormality. A chi-square test was used to examine PEMAT-AV actionability as a dichotomous variable (actionable vs not) due to a high number of 0 values.

### Ethical Considerations

This study was submitted to the University of Florida Institutional Review Board, which determined it to be exempt from full board review because all information was obtained from publicly available content. Given that only publicly available data were collected and analyzed, there was no need to safeguard participant information.

## Results

### Video Quality Classification

**Figure 1.**
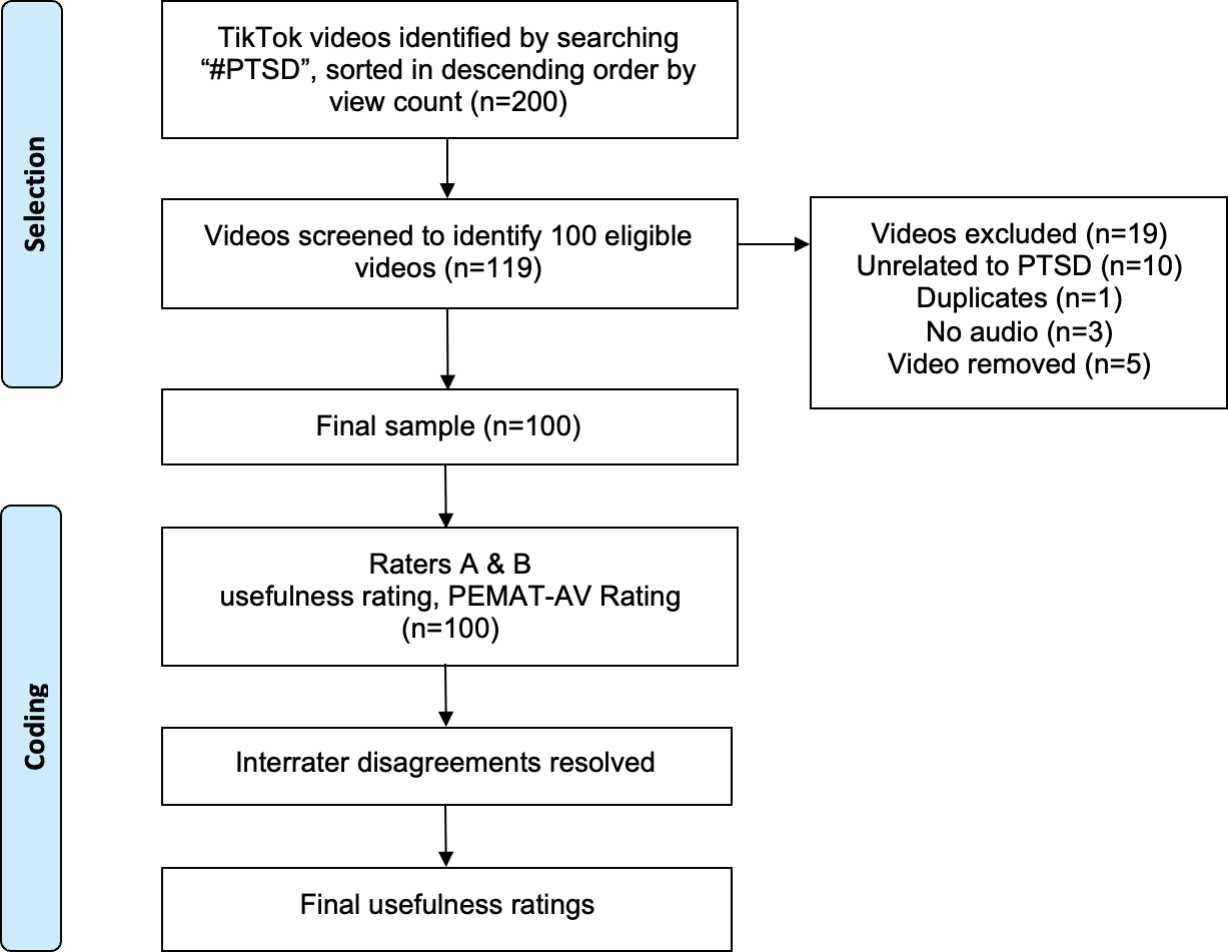
Outline of search, selection, and rater coding procedures for top 100 #PTSD TikTok videos. Video selection was completed by author BR and coding was completed independently by authors LCJ and RN. In the rare event of interrater disagreements, BR provided resolution. PEMAT-AV: Patient Education Materials Assessment Tool for Audiovisual Materials; PTSD: posttraumatic stress disorder.

Of the 100 included videos, 29 were classified as useful, 59 were classified as personal experience (subjective experience without outright inaccuracies), and 12 were classified as misleading. Examples of content related to each category are described in Textbox 1. Figure 1 outlines the procedures used for search, selection, and rater coding.

Textbox 1. **Descriptions of example #PTSD TikTok videos by usefulness classification.**

Useful (n=12)

- An infographic containing two pie charts is superimposed over an image of a sunset, and lo-fi music is playing. One pie chart is titled, “What people think PTSD is,” and consists of one category labeled, “Not being able to move on after a traumatic event.” The other pie chart is titled, “What PTSD actually is” and is comprised of a number of equally proportioned categories labeled as “unwanted memories,” “negative self-image,” “hypervigilance,” “emotional distress,” “sense of distrust,” “intrusive thoughts,” “sense of threat,” “avoidance/isolation,” “memory problems,” “anger, guilt and shame,” “anxiety/depression,” “excessive blame,” “dissociation,” “easily scared,” “flashbacks,” “sleeping problems,” and “self-destructive behaviors.”
- A self-identified clinical psychologist explains the differences in diagnostic criteria between posttraumatic stress disorder (PTSD) and borderline personality disorder.

Personal experiences (n=59)

- A selfie-style video of a man saying the following: “Three things you should not do to a Veteran with PTSD: 1) Walk up behind them. You’re going to end up startling them, and you’re going to end up getting hurt. And it doesn’t matter who you are. 2) Scare pranks. You may just have the same result as the first one we just talked about, which, you’re going to get hurt. Second because some of us take hours to get our head straight for the day, and being scared like that puts us back into that state of mind that we work so hard to get out of. 3) Ask about combat experiences. If we haven’t told you already about our military experiences and what we experienced while we were deployed, the chances of us telling you are slim to none. Mainly because we don’t want to remember. A lot of us lost friends and family. If we do decide to share, nine times out of ten we put ourselves into a thousand-yard stare where we’re re-living the situation all over again. The more you know.”
- A woman films herself waking up and having “bad anxiety” which quickly triggers her PTSD. A “flashback” is preceded by eye twitching. She states that she is grounding herself in reality by touching the counter and looking around for visible proof that she is not physically back at the scene of a trauma.

Misleading (n=29)

- Clips from video games are accompanied by sound effects associated with each game. Superimposed text reads, “Sounds that will give gamers PTSD.” As the game-specific sound effects play, superimposed text denotes the name of each video game.
- A self-identified clinical psychologist discusses “what people don’t understand about complex trauma.” While some accurate information is presented, inaccuracies include “sensitivities to sounds like breathing and chewing” and “maladaptive daydreaming” with a “rich fantasy life.”

### Characteristics of Outliers

A total of 3 videos were removed from primary analyses due to extreme metadata values. Of these, one (classified as personal experience) exhibited extreme values across all metadata variables, having earned 38.6 million views (7.25 SDs above mean), 72 million likes (8.2 SDs above mean), 44,000 shares (3.6 SDs above mean), and 26,100 comments (3.58 SDs above mean). Qualitative notes indicate that the video contains a depiction of a service animal helping a trauma survivor cope with navigating the physical site where the event occurred. The other 2 videos varied with respect to which variable was extreme, with one having only a high number of comments (11.8 million, 7.25 SDs above mean, personal experience, content depicts a personal trauma narrative), and the other having only a high number of shares (83,300, 7.3 SDs above mean, misleading, content depicts a joke).

### Video Metadata and Usefulness Group

**Figure 2.**
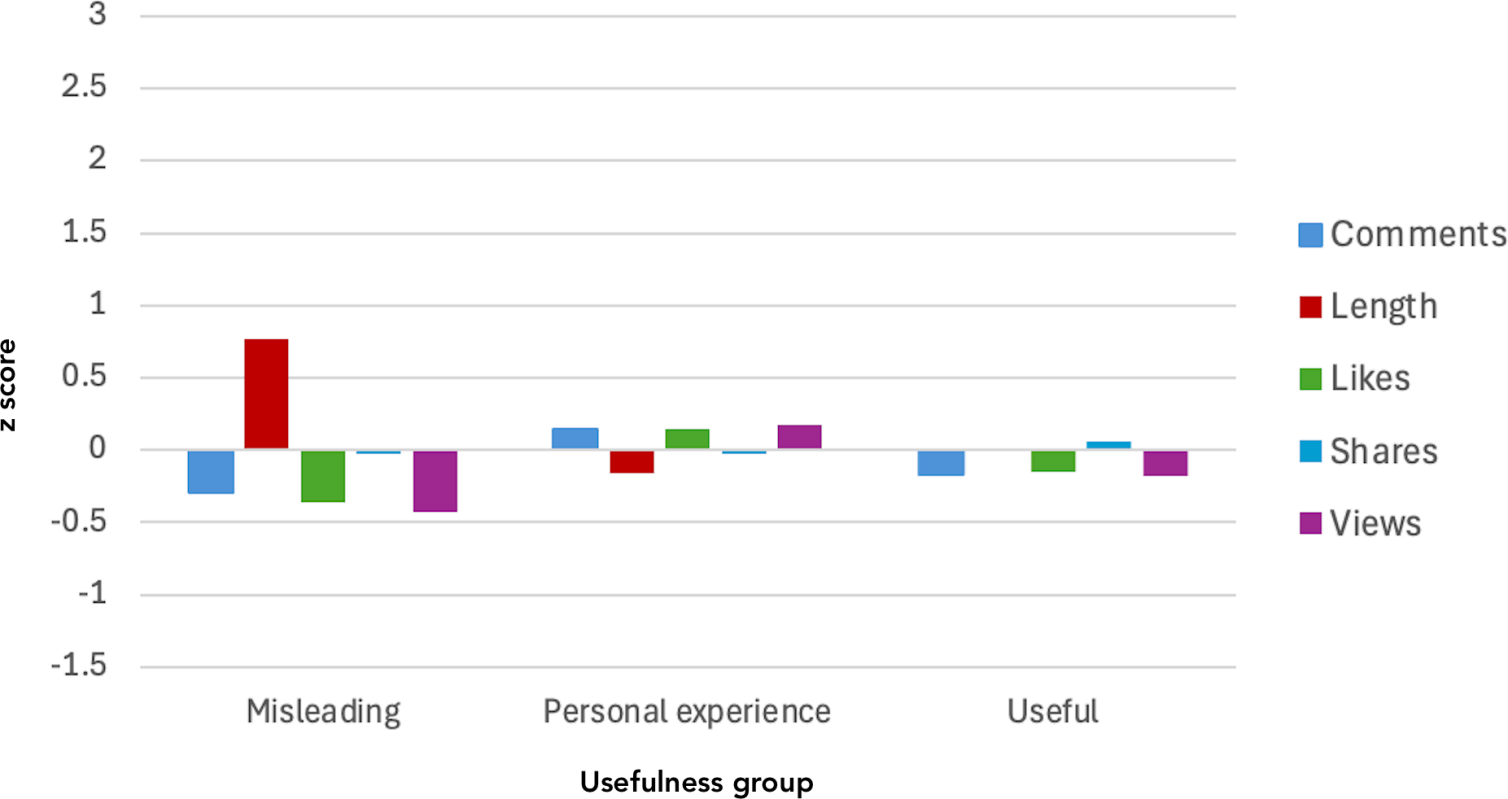
Metadata by top 100 #PTSD TikTok video usefulness group.

Results are presented in Table 2 and Figure 2. A significant group difference was observed for video length (*F*_2,30.7_=5.34; *P*=.01) such that useful videos tended to be longer (mean 88.7, SD 63.1 seconds) than personal experience videos (mean 42.7, SD 44.5 seconds). No group differences were observed across the number of views (*χ*^2^_2_=4.42; *P*=.11), likes (*χ*^2^_2_=5.19; *P*=.07), shares (*χ*^2^_2_=0.94; *P*=.63), or comments (*χ*^2^_2_=2.45; *P*=.29)

**Table 2.** Raw top 100 #PTSD TikTok videos’ metadata.

Characteristics	Misleading (n=29)	Personal experience (n=59)	Useful (n=12)	Overall group (N=100)
Video length (seconds), mean (SD)	50.28 (48.91)	42.69 (44.45)	88.67 (63.14)	50.41 (49.9)
Views, mean (SD; range)	1,981,552 (2,823,886; 25,100-11,200,000)	3,716,873 (5,947,155; 12,800-38,600,000)	737,475 (811,699; 33800-2,500,000)	2856102 (4925371; 12800-38600000)
Likes, mean (SD; range)	273,956 (423,923; 2,251-1,500,000)	520,741 (1,017,290; 1867-579,100)	99,070 (160,473; 1,867-579,100)	398,572 (827,222; 801-7,200,000)
Shares, mean (SD; range)	5374 (16,265; 125-83,300)	4457 (7384; 9-44,000)	4391 (8,355; 43-29,600)	4714 (10,709; 9-83300)
Comments, mean (SD; range)	2266 (2932; 1-12,000)	4244 (7862; 27-49,300)	1493 (1790; 15-5,343)	3340 (6343; 1‐49300)

### Communication Effectiveness, Usefulness Group, and HCP Status

Results are presented in Table 3. There were no group differences across any measures of communication effectiveness, including PEMAT-AV total score (*P*=.06), PEMAT-AV understandability (*P*=.12), or the presence of PEMAT-AV actionability points (*P*=.14). A model with self-identified HCP status and usefulness classification as predictors of communication effectiveness found no significant effect for PEMAT-AV total (*P*=.38). Self-identified HCPs were responsible for posting 32% (4/12) of misleading content, 14% (9/59) of personal experience content, and 67% (19/29) of useful content, a significant difference between HCPs and non-HCPs (*χ*^2^_2_=15.19; *P*<.001).

**Table 3.** Communication effectiveness from top 100 #PTSD TikTok videos and creator status by usefulness group.

	Misleading (n=12)	Personal experience (n=59)	Useful (n=29)	*P* value
PEMAT-AV^a^ understandability score, n (%)	58.24 (12.98)	52.63 (12.14)	55.13 (13.05)	.12
PEMAT-AV actionability score >0, %	10	12	33	.14
PEMAT-AV total score, n (%)	46.85 (9.54)	42.11 (10.11)	45.59 (8.37)	.38
Self-identified HCPs^b^, %	32	14	67	<.001

a PEMAT-AV: Patient Education Materials Assessment Tool for Audiovisual Materials.

b HCP: health care professional.

### Representations of Trauma Type and *DSM-5* Symptoms

Multimedia Appendix 1 shows the frequency of trauma types and sources represented in our sample, as well as the *DSM-5* symptoms that were mentioned. Of note, the types of traumatic event do not add to 100% because not every creator reported a type of traumatic event.

## Discussion

### Principal Findings

We found that approximately one-third of the most popular TikTok content related to PTSD contained inaccurate information. The degree to which PTSD-related information was accurate was not associated with its understandability, actionability, or user engagement. Self-identified HCPs were more likely than non-HCPs to post useful information; however, HCPs still accounted for 32% of the content classified to be misleading.

Our findings were roughly consistent with previous studies of mental health–related TikTok content accuracy [10,25]. Studies of other medical conditions reveal variable quality depending on diagnosis: for example, information related to diabetes was generally determined to be of acceptable quality, although lacking in comprehensiveness to meet patient needs [26], and information related to mitral valve regurgitation was generally determined to be factually accurate but lacking in clarity of explicit actionable steps [27]. Of note, TikTok videos are quite short compared to other video-based platforms like YouTube, and it is possible that pressure to fit information in a smaller space leaves less room for misinformation than has been reported to occur there [28]. The content reviewed for this study generally appeared to be informally generated and created by nonexperts or HCPs, so although there was a lack of “actionable steps,” this may reflect reluctance to offer advice, which may be considered a positive finding. In addition, the dangers posed by inaccurate information spread may vary according to the characteristics of the condition in question, such as the observability of pathognomonic signs, impact on insight, and implications of meeting diagnostic criteria (eg, prognosis).

In the case of PTSD, this could take the following forms. The misattribution of symptoms with respect to traumatic origin (a problem with specificity) could contribute to both underidentification with trauma disorders (ie, attributing PTSD symptoms to some other cause, such as ADHD), or overidentification with trauma disorders (ie, misattributing nonspecific symptoms to the experience of an adverse event). Previous work shows that group identification (in vs out) is a primary theme of PTSD-specific discussion on internet forums and may serve to reproduce team-based military environments or facilitate “safe” social engagement despite characteristic feelings of detachment or estrangement from other people in general (Criterion D) [11].

Research has shown that aspects of online behavior can become incorporated into users’ identities [29]. Once a user is engaged with diagnosis-related content, inaccurate information about prognosis (ie, the common negative belief that people with PTSD are “permanently broken”) could endanger one’s sense of control, hope for the future, and engagement with treatment. In our view, poor expectations for prognosis are likely the biggest risk from inaccurate information spread about PTSD.

A nonjudgmental approach to practicing clinical psychology in the modern era must acknowledge the very real risks of health misinformation, but also consider the benefits of social media use in providing free, health-related information, and the development of communities around shared symptoms and diagnoses. One such outcome is a sense of support and validation gained through discussions of shared experiences. This could look like a series of reciprocal social interactions (ie, discussions in the comments section under posts), or the building of a community around a particular experience, trend, or content creator. Such community building may function to serve the additional themes of PTSD-specific online discussion identified by Stana et al [11]: overcoming stigma, providing and receiving informational support, and embracing conflict through humor.

For patient populations particularly susceptible to barriers to care related to stigma, changes in diagnostic accuracy over time, and the tendency for symptoms to interrupt engagement with health care information presented in other ways, the delivery of mental health–related information via TikTok is an invaluable resource. For example, a woman in her 60s with a history of learning problems (a problem historically underdiagnosed in girls) and trauma exposure may have never, before accessing TikTok, accessed the information necessary to understand that her day-to-day difficulties may relate to either of these risk factors. If HCPs are acquainted with the digital landscapes inhabited by their patients, these internet resources can be leveraged to extend providers’ impact between therapy sessions by supporting patients’ critical consumption of this information and even encouraging the discussion of internet content in session.

It is important to consider that TikTok’s endless feed represents a massive publicly available source of human behavioral data that may not fit neatly into established categorical systems. It is possible that nonspecific symptoms “overly attributed” to a diagnostic category could reflect a spectrum of patient experiences not captured with longstanding frameworks. The phrase “complex trauma” was specifically mentioned in about one-fourth of our sample. Complex trauma refers to prolonged and repeated exposure to trauma (as opposed to that which occurs via discrete events), for example, exposure to domestic violence, child abuse, human trafficking, and prisoner of war or refugee status [30]. Early research definitions of complex trauma stipulated that the victim is held in a state of physical or emotional captivity by a perpetrator [31].

Complex trauma is not well-captured by current PTSD diagnostic criteria, and the degree to which it may confer a proposed PTSD phenotype known as complex PTSD is the subject of ongoing debate [32,33]. Proponents of a distinct complex PTSD phenotype maintain that diffuse effects of prolonged, repeated trauma on affect regulation, behavioral dysregulation, self-concept, and development (biological and relational) are inefficiently captured and treated by an approach that assigns multiple highly comorbid diagnoses, such as mood and attachment disorders, ADHD, eating disorders, and sleep disorders. Future work assessing trauma-related content on social media should specifically investigate discussion of complex trauma.

Our study had several strengths. To the best of our ability, we mitigated the effect of our own demographic characteristics on algorithmically driven results of a strategic query by creating a new account and not engaging with features known to impact video recommendations. Also, all human-rated variables were determined via agreement between two independent raters familiar with clinical research related to PTSD, with a third rater resolving rare disagreements between them.

However, our findings should be interpreted in the context of known limitations. Our search approach was strategically intended to capture popular PTSD-related content, but it is not representative of the typical user experience. TikTok uses a proprietary algorithm that is not available to the public but is widely believed to heavily weigh greater user engagement, based on both user experience as well as a leaked internal document confirmed to be a training document for nontechnical employees [34]. According to TikTok’s website, the application of its algorithm also differs between users, with the relative weight of indicators of interest, such as video completion or relative geographic proximity of creator and viewer, varying based on assumed importance to the individual user. Second, information about how TikTok’s algorithms deliver content through search is not publicly available, and therefore, we cannot know for sure whether these videos represent the most engaged-with.

### Conclusions

Approximately one-third of popular PTSD-related TikTok content contained inaccurate information. Accuracy was not linked to content understandability, actionability, or engagement. While self-identified HCPs were more likely to share useful information than nonproviders, they still produced nearly one-third of the misleading content. The democratization of mental health information provides opportunities for individuals who would not otherwise access high-quality care to learn about the experiences and trajectories of others, including free, crowd-sourced tips for managing symptoms. In contrast to other sources of online health information, including other social media platforms, TikTok’s primary method of media delivery through an algorithmically determined, continuously adaptive stream of recommended content has the potential to inundate individuals with nonspecific mental health information without users having explicitly searched for it, as might otherwise happen on other sites like YouTube or Facebook. This may, in turn, impact patients’ interpretations of their symptoms and ultimately their clinical presentation. Future studies should assess how trends within social media content related to mental health conditions may impact patients’ engagement with assessment and treatment within the American health care system.

## Supplementary material

10.2196/71209 Multimedia Appendix 1 Percentages of trauma and posttraumatic stress disorder symptoms described in the top 100 #PTSD TikTok videos.

## Abbreviations

ADHD: attention-deficit hyperactivity disorder
*DSM-5*: *Diagnostic and Statistical Manual of Mental Disorders, Fifth Edition*
FYP: For Your Page
HCP: health care professional
PEMAT-AV: Patient Education Materials Assessment Tool for Audiovisual Materials
PTSD: posttraumatic stress disorder

## References

[1] Woolard A, Paciente R, Munro E (2024). #TraumaTok-TikTok videos relating to trauma: content analysis. JMIR Form Res.

[2] Milton A, Ajmani L, DeVito MA, Chancellor S (2023). “I see me here”: mental health content, community, and algorithmic curation on tiktok.

[3] Papacharissi Z (2011). A Networked Self: Identity, Community, and Culture on Social Network Sites.

[4] Bhandari A, Bimo S TikTok and the algorithmized self: a new model of online interaction. http://spir.aoir.org.

[5] Trauma response. Google Trends.

[6] Palus S (2021). Why TikTok is so obsessed with labeling everything a trauma response. Slate Magazine.

[7] Stop your depression by being happy. we know this. TikTok.

[8] Giedinghagen A (2023). The tic in TikTok and (where) all systems go: mass social media induced illness and Munchausen’s by internet as explanatory models for social media associated abnormal illness behavior. Clin Child Psychol Psychiatry.

[9] Zea Vera A, Bruce A, Garris J (2022). The phenomenology of tics and tic-like behavior in TikTok. Pediatr Neurol.

[10] Yeung A, Ng E, Abi-Jaoude E (2022). TikTok and attention-deficit/hyperactivity disorder: a cross-sectional study of social media content quality. Can J Psychiatry.

[11] Stana A, Flynn MA, Almeida E (2017). Battling the stigma: combat veterans’ use of social support in an online PTSD forum. International Journal of Men’s Health.

[12] Zheng DX, Ning AY, Levoska MA, Xiang L, Wong C, Scott JF (2021). Acne and social media: a cross-sectional study of content quality on TikTok. Pediatr Dermatol.

[13] Basch CH, Meleo-Erwin Z, Fera J, Jaime C, Basch CE (2021). A global pandemic in the time of viral memes: COVID-19 vaccine misinformation and disinformation on TikTok. Human Vaccines & Immunotherapeutics.

[14] Russell AM, Davis RE, Ortega JM, Colditz JB, Primack B, Barry AE (2021). #Alcohol: portrayals of alcohol in top videos on TikTok. J Stud Alcohol Drugs.

[15] Leong AY, Sanghera R, Jhajj J, Desai N, Jammu BS, Makowsky MJ (2018). Is YouTube useful as a source of health information for adults with type 2 diabetes? A South Asian perspective. Can J Diabetes.

[16] (2023). How to use hashtags on TikTok. #Hashtag Expert.

[17] TikTokPy. Python Package Index.

[18] TikTok scraper. DEV Community.

[19] Shoemaker SJ, Wolf MS, Brach C (2014). Development of the Patient Education Materials Assessment Tool (PEMAT): a new measure of understandability and actionability for print and audiovisual patient information. Patient Educ Couns.

[20] Sood A, Sarangi S, Pandey A, Murugiah K (2011). YouTube as a source of information on kidney stone disease. Urology.

[21] Kumar N, Pandey A, Venkatraman A, Garg N (2014). Are video sharing web sites a useful source of information on hypertension?. J Am Soc Hypertens.

[22] (2013). Diagnostic and Statistical Manual of Mental Disorders.

[23] Blom G (1954). Transformations of the binomial, negative binomial, Poisson and χ^2^ distributions. Biometrika.

[24] Mishra S, Diesner J Detecting the correlation between sentiment and user-level as well as text-level meta-data from benchmark corpora.

[25] Lookingbill V, Mohammadi E, Cai Y (2023). Assessment of accuracy, user engagement, and themes of eating disorder content in social media short videos. JAMA Netw Open.

[26] Kong W, Song S, Zhao YC, Zhu Q, Sha L (2021). TikTok as a health information source: assessment of the quality of information in diabetes-related videos. J Med Internet Res.

[27] Cui N, Lu Y, Cao Y, Chen X, Fu S, Su Q (2024). Quality assessment of TikTok as a source of information about mitral valve regurgitation in China: cross-sectional study. J Med Internet Res.

[28] Morra S, Collà Ruvolo C, Napolitano L (2022). YouTube^TM^ as a source of information on bladder pain syndrome: a contemporary analysis. Neurourol Urodyn.

[29] Kirmayer LJ, Raikhel E, Rahimi S (2013). Cultures of the internet: identity, community and mental health. Transcult Psychiatry.

[30] Courtois CA (2004). Complex trauma, complex reactions: assessment and treatment. Psychotherapy: Theory, Research, Practice, Training.

[31] Herman JL (1992). Complex PTSD: a syndrome in survivors of prolonged and repeated trauma. J Trauma Stress.

[32] Cook A, Spinazzola J, Ford J (2005). Complex trauma. Psychiatr Ann.

[33] Resick PA, Bovin MJ, Calloway AL (2012). A critical evaluation of the complex PTSD literature: implications for DSM-5. J Trauma Stress.

[34] Smith B (2021). How TikTok reads your mind. The New York Times.

