# Supplementary figures and images for: Posttraumatic Stress Disorder Content on TikTok: Cross-Sectional Analysis of Popular #PTSD Posts
Source: Online J Public Health Inform. 2025 Sep 10;17:e71209. doi: 10.2196/71209 (PMC12422738; doi:10.2196/71209)

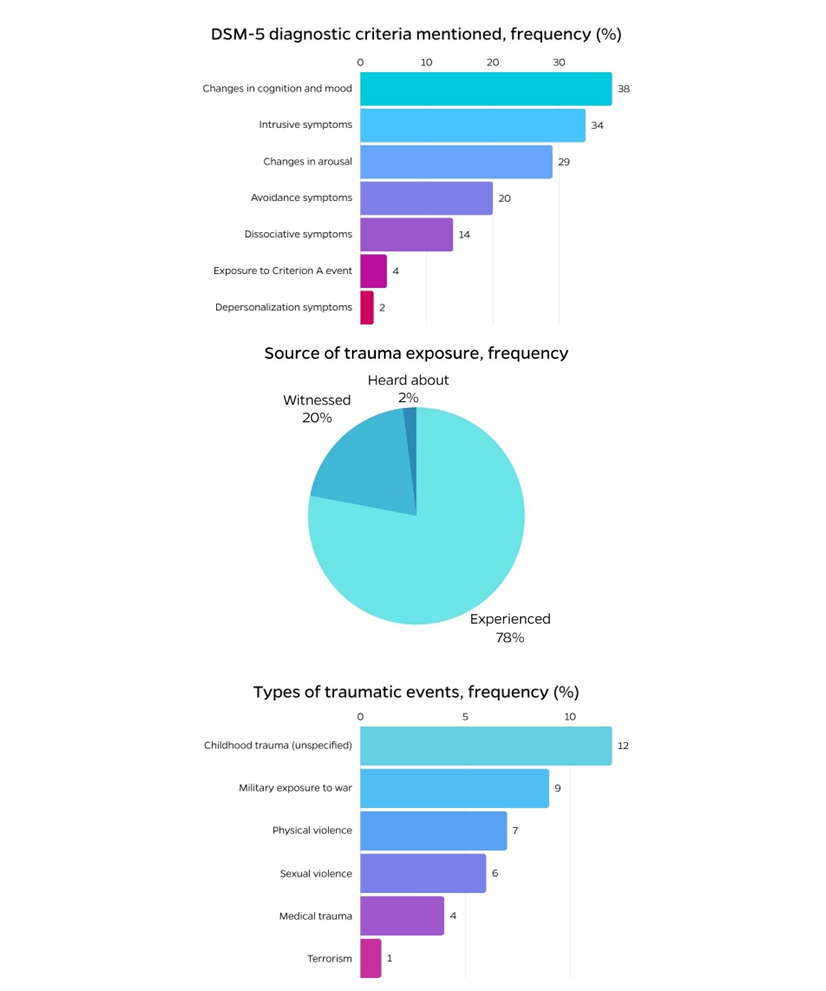

Supplement: Multimedia Appendix 1 [file ojphi-v17-e71209-s001.png]
